# RNA‐Peptide nanoplexes drug DNA damage pathways in high‐grade serous ovarian tumors

**DOI:** 10.1002/btm2.10086

**Published:** 2018-01-19

**Authors:** Erik C. Dreaden, Yi Wen Kong, Mohiuddin A. Quadir, Santiago Correa, Lucia Suárez‐López, Antonio E. Barberio, Mun Kyung Hwang, Aria C. Shi, Benjamin Oberlton, Paige N. Gallagher, Kevin E. Shopsowitz, Kevin M. Elias, Michael B. Yaffe, Paula T. Hammond

**Affiliations:** ^1^ Koch Institute for Integrative Cancer Research Massachusetts Institute of Technology Cambridge MA 02139; ^2^ Dept. of Chemical Engineering Massachusetts Institute of Technology Cambridge MA 02139; ^3^ Dept. of Biology Massachusetts Institute of Technology Cambridge MA 02139; ^4^ Dept. of Biological Engineering Massachusetts Institute of Technology Cambridge MA 02139; ^5^ Dept. of Obstetrics, Gynecology, and Reproductive Biology, Brigham and Women's Hospital Harvard Medical School Boston MA 02215; ^6^ Div. of Acute Care Surgery, Trauma, and Surgical Critical Care, Dept. of Surgery, Beth Israel Deaconess Medical Center Harvard Medical School Boston MA 02215; ^7^ Institute for Soldier Nanotechnologies Massachusetts Institute of Technology Cambridge MA 02139; ^8^ Present address: Wallace H. Coulter Dept. of Biomedical Engineering Georgia Institute of Technology and Emory University, Atlanta, GA 30322; Dept. of Pediatrics, Aflac Cancer and Blood Disorders Center, Children's Healthcare of Atlanta, Emory University School of Medicine Atlanta GA 30322; ^9^ Present address: Dept. of Coatings and Polymeric Materials North Dakota State University Fargo ND 58108; ^10^ Present address: Faculty of Medicine University of British Columbia BC V1Y 1T3 Canada

**Keywords:** chemosensitization, DNA damage, nanomedicine, ovarian cancer, polymer engineering, RNA interference

## Abstract

DNA damaging chemotherapy is a cornerstone of current front‐line treatments for advanced ovarian cancer (OC). Despite the fact that a majority of these patients initially respond to therapy, most will relapse with chemo‐resistant disease; therefore, adjuvant treatments that synergize with DNA‐damaging chemotherapy could improve treatment outcomes and survival in patients with this deadly disease. Here, we report the development of a nanoscale peptide‐nucleic acid complex that facilitates tumor‐specific RNA interference therapy to chemosensitize advanced ovarian tumors to frontline platinum/taxane therapy. We found that the nanoplex‐mediated silencing of the protein kinase, MK2, profoundly sensitized mouse models of high‐grade serous OC to cytotoxic chemotherapy by blocking p38/MK2‐dependent cell cycle checkpoint maintenance. Combined RNAi therapy improved overall survival by 37% compared with platinum/taxane chemotherapy alone and decreased metastatic spread to the lungs without observable toxic side effects. These findings suggest (a) that peptide nanoplexes can serve as safe and effective delivery vectors for siRNA and (b) that combined inhibition of MK2 could improve treatment outcomes in patients currently receiving frontline chemotherapy for advanced OC.

## 1 INTRODUCTION

RNA interference (RNAi) therapy is a powerful treatment strategy that can be used to transiently deplete disease‐causing proteins.^1^ While promising, systemic administration of RNAi therapeutics such as small interfering RNA (siRNA) in patients is challenging due to rapid urinary clearance, nuclease degradation, and inefficient tissue, cellular, or cytosolic delivery. Formulation strategies for siRNA based on phospholipid^2^, ^3^ and lipid‐like^4^, ^5^ carriers have conferred therapeutic benefits in animal disease models and in clinical trials^6^, ^7^; however, efficient tissue‐specific delivery beyond the liver, and avoidance of dose‐limiting side effects remains a key challenge for the field. In contrast to antisense oligonucleotide therapies that have been FDA approved since 1998, no siRNA‐based therapies have been approved to‐date.

**Figure 1:**
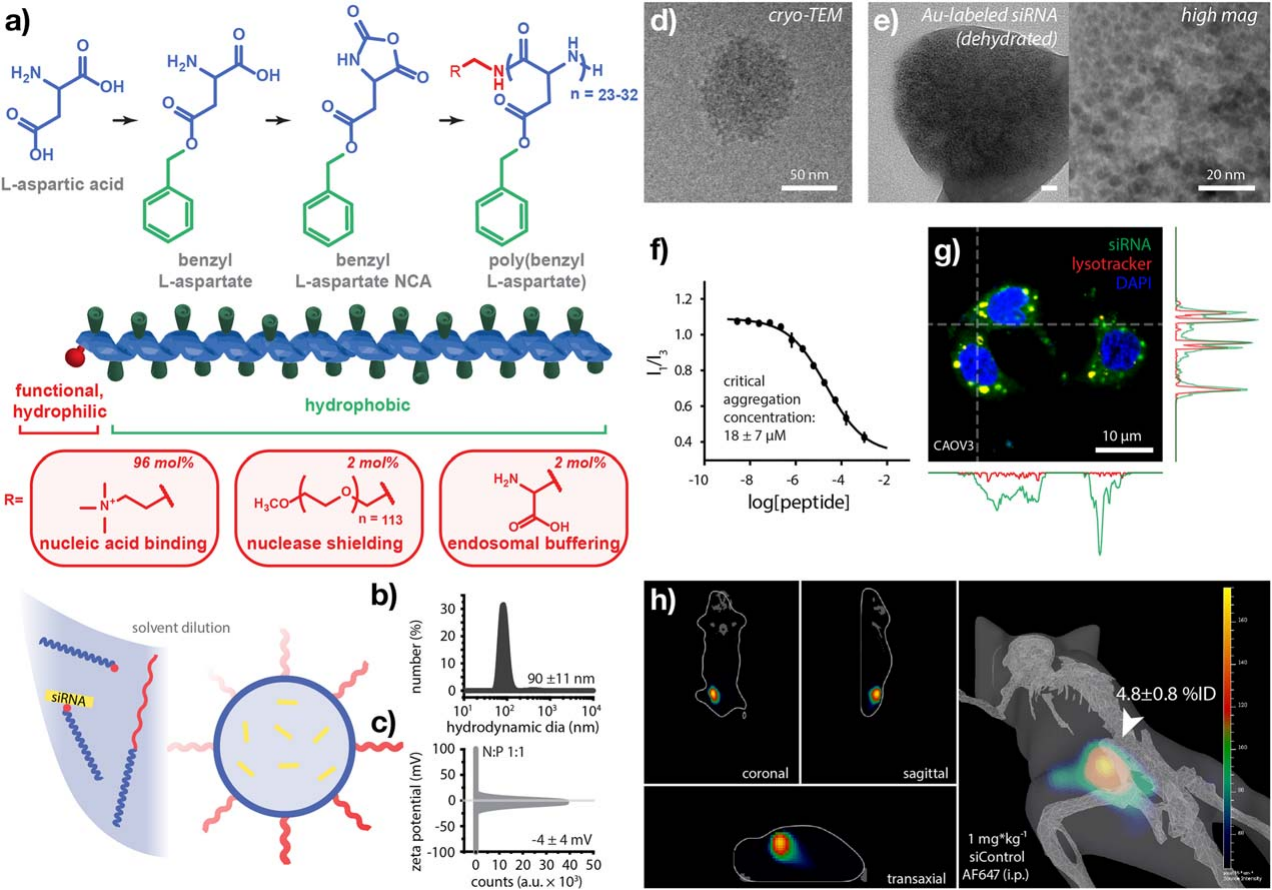
Rational design of a synthetic peptide nanoplex for RNA interference therapy. (a) Synthetic scheme to obtain end‐functional *N*‐carboxyanhydride (NCA) polymers via ring‐opening polymerization of benzyl L‐aspartate, yielding peptides that facilitate nucleic acid binding, nuclease shielding, or cytosolic delivery. Solvent dilution in the presence of siRNA produces (b) nanometer‐scale polyelectrolyte complexes that are (c) net‐neutral in charge as measured by dynamic light scattering (DLS). (d) Cryogenic transmission electron microscopy (cryo‐TEM) of RNA‐peptide nanoplexes indicating spherical morphology in the hydrated state and (e) electron microscopy of dehydrated nanoparticles formed using gold nanoparticle‐labeled siRNA showing homogeneous siRNA distribution throughout the ionic complex core. (f) Nanoplex stability (critical aggregation concentration) as measured by pyrene fluorescence assay. (g) Live‐cell confocal fluorescence microscopy of nanoplex‐mediated cell transfection illustrating cytosolic delivery of siRNA (green) relative to acidic organelles (red) and nuclei (blue). (h) Fluorescence imaging tomography (FLIT) of nanoplex‐delivered siRNA distributed throughout an OVCAR3 hind‐flank xenograft tumor model following intraperitoneal administration. Data in (b–h) were obtained at an N‐to‐P ratio of 1. (f) 335 nm ex, 373 nm em (I1), 384 nm em (I3). (g) 20 nM siRNA (AllStars negative control, 3′‐Alexa Fluor 488), vesicles (Lysotracker Red DND‐99), and nuclei (Hoechst 34580) at 1 hr. (h) 1 mg/kg siRNA (AllStars negative control, 3′‐Alexa Fluor 647), 640/700 nm ex/em at 24 hr. Images in (h) were obtained in a transillumination configuration which limits observable signal, here, to the hind flanks. Error represents (b,c) SD of three technical, (f) SD of three biological, and (h) SD of four biological replicates

Polymer‐based drug carriers^8^, ^9^, ^10^, ^11^, ^12^, ^13^, ^14^ represent a versatile and potentially safe alternative to lipidic oligonucleotide drug carriers, and related architectures have been utilized in a range of clinically approved drug formulations for more than three decades. Due to their modular nature, polymers can be engineered to package, protect, and deliver siRNAs to tumors, and can afford therapeutic nucleic acids access to the cytosol where they can inhibit the expression of oncogenic and pro‐survival genes, among others. Recently, we demonstrated that a family of polypeptides synthesized by *N*‐carboxyanhydride (NCA) ring‐opening polymerization^15^, ^16^, ^17^ can serve as modular scaffolds for the delivery of both small molecule drugs^16^, ^18^, ^19^ and nucleic acids.^20^ Previous strategies to formulate siRNA using synthetic polypeptides have focused largely on the formation of electrostatic complexes with cationic block copolymers.^21^ The resulting systems facilitate efficient complexation of nucleic acids; however, highly charged polycationic blocks can be challenging to engineer such that siRNAs are efficiently released from complexes entrapped within endosomal compartments. Design strategies for nucleic acid carriers thus require a balance between high valency of charge (complexation) and intracellular release or cytotoxicity. In place of a more traditional diblock copolymer with a charged or neutral water‐soluble block, we have engineered a family of NCA polymers derived from short oligopeptides that spatially isolate varying delivery functions within the final nanoparticle (Figure 1a). By lowering the charge density of the positively charged component and introducing other intermolecular interactions, here we can employ a more dynamic polyelectrolyte complex system than the typical ionically crosslinked matrix. Supramolecular NCA architectures in which siRNA is electrostatically complexed within a dynamically complexed core could thus provide new opportunities for tumor‐targeted siRNA delivery in vivo.

Despite many encouraging advances in addressing other common cancers such as breast and prostate cancer, there has been little to no improvement in the survival rate of ovarian cancer (OC) patients over the past 30 years.^22^ The 5‐year survival rates for the disease have hovered at approximately 30–40% in the past several decades, and OC is now the fifth most lethal cancer for women.^23^ Today, nearly all patients diagnosed with epithelial OC will be treated with DNA‐damaging platinum‐based chemotherapy.^24^ Particularly for advanced serous OC, surgical debulking followed by platinum therapy is the treatment of choice. Of these patients, approximately 20–30% will not respond to the initial therapy, and of the remaining patients, approximately 70% eventually relapse with a platinum‐resistant form of the disease.^25^ Methods to sensitize aggressive or recurrent ovarian tumors to platinum chemotherapy could thus improve response rates and overall survival following diagnosis, which most often occurs in advanced stages. Recently, we identified a p38MAPK/MK2‐dependent DNA damage response pathway that is selectively required for survival of cells deficient in the tumor suppressor, p53.^26^, ^27^, ^28^, ^29^ We found^28^ that loss of the protein kinase, MK2, profoundly sensitized non‐small cell lung cancers to DNA damaging chemotherapy in vivo, resulting from corresponding loss of cell cycle checkpoint maintenance and subsequent mitotic catastrophe.^27^ Importantly, we observed that loss of this response pathway is inconsequential in normal (i.e., p53‐proficient) cells, thus limiting chemosensitization to tumor cells harboring genetic mutations in the p53 tumor suppressor protein.^26^, ^27^, ^28^, ^29^ Given that TP53 mutation and/or loss is a hallmark of high‐grade serous OC and is observed in >96% of patients,^30^ we seek here to develop this therapeutic approach for the treatment of OC.

Although MK2 is a promising drug target due to its role in both cancer and inflammation, commercial development of small molecule inhibitors against the protein have experienced little clinical success due in part to the kinase's shallow binding pocket and sequence homology with other physiologically important kinases (e.g., MK3, MK5, MNK1, MNK2). Furthermore, MK2 plays an important role in the immune response.^31^ Thus, highly selective *and* tissue‐specific silencing of this “undruggable” target in tumors represents a novel and unmet clinical need, and provides the opportunity to utilize engineered nanocarriers to deliver chemosensitizing siRNA with concurrent cytotoxic chemotherapy for the treatment of advanced serous OC. We hypothesize that RNAi of MK2 mediated by synthetic peptide nanoplexes may sensitize p53‐deficient ovarian tumors to DNA damaging chemotherapy and improve treatment outcomes in OC patients receiving frontline platinum/taxane therapy; here, we demonstrate their therapeutic performance in vitro and in orthotopic mouse models of late stage, high‐grade serous OC.

## 2 MATERIALS AND METHODS

### 2.1 Materials

Silencer Select siRNA targeting human *MAPKAPK2* (s569; Thermo Fischer); sense: GGAUCAUGCAAUCAACAAATT, antisense: UUUGUUGAUUGCAUGAUCCAA. Antibodies included: p‐P38 (CST 4511S), MK2‐WB (CST 3042), vinculin (Abcam ab18058), MK2‐IHC (Bioworld BS3766), Ki‐67 (Abcam ab16667), cleaved caspase‐3 (CST 9661), PAX8 (Proteintech 10336‐1‐AP), and WT1 (Abcam ab89901).

### 2.2 Nanoplex assembly and analysis

Peptides (96:2:2 mol% cation:helper:PEG; CHCl_3_; N:P = 1) were aliquoted into sterile chromatography vials, dried under N_2_, and reconstituted with DMSO. Aqueous siRNA solution was added to >60% v/v and the mixture was diluted 20‐fold with a 50% ethanol : water (ca. 0.5 mL/min) under sonication at RT. Peptide‐siRNA complexes were purified by dialysis (3.5‐5 kDa) against ultrapure water at 4°C and diluted in 1× PBS immediately prior to injection by dropwise addition of 10× PBS buffer with sonication at RT. siRNA encapsulation efficiency was 75 ± 10% as measured using fluorescently labeled siRNA (AllStars Negative Control siRNA, Alexa Fluor 647; Qiagen, Hilden, Germany).

Photon correlation spectroscopy and laser Doppler electrophoresis measurements were carried out in molecular biology grade deionized water using a Malvern Zetasizer Nano ZS90 particle analyser (λ = 633 nm, material/dispersant RI 1.590/1.330). Critical aggregation concentration was determined as described previously^32^ using a four‐parameter logistic fit (λ_ex_ = 335 nm, λ_em,I1_ = 373 nm, λ_em,I3_ = 384 nm; n = 3 replicates).

### 2.3 In vitro studies

OVCAR8 cells were a gift from S. Bhatia (MIT). OVCAR3, Caov3, and TOV‐112D cells were obtained from ATCC. Cells were subcultured in DMEM supplanted with 10% fetal bovine serum and penicillin/streptomycin, or the supplier's recommended basal medium, in a 5% CO_2_ humidified atmosphere. Lines were screened for mycoplasma (Swanson Biotechnology Center, Cambridge, MA) and authenticated by short tandem repeat analysis (Promega) both after viral transduction and prior to tumor induction.

In vitro knockdown was assessed in COV362 cells following forward transfection in OPTI‐MEM media for 72 hr (10 nM siRNA) using nanoplexes assembled as described above or Lipofectamine RNAiMAX (Life Technologies) as formulated per the manufacturer's recommended conditions. shRNAs were designed using the Cold Spring Harbor web portal^33^ and 97mer oligonucleotides were used as templates for PCR using miR‐30 shRNA amplification primers. The PCR product was cloned into the miR30‐based retroviral vector pMLP, a kind gift from M. Hemann (MIT). The oligonucleotide for targeting MK2 (human, NM_032960.2) was 5′‐TGCTGTTGACAGTGAGCGAAGCGAAATTGTCTTTACTAAATAGTGAAGCCACAGATGTATTTAGTAAAGACAATTTCGCTCTGCCTACTGCCTCGGA‐3′.

Colony formation assays were performed following treatment with vehicle, cisplatin, or paclitaxel for 4 hr. TOV112D cells were then washed, trypsinized, and re‐plated at a concentration of 1,000 cells/well for mock‐treated groups and 10,000 cells/well for chemotherapy‐treated groups in a 6‐well dish. After 10 days, cells were fixed and stained with modified Wright‐stain (Sigma‐Aldrich). Colonies were enumerated and surviving fractions were determined by normalization with vehicle treated cells.

Tissue microarrays were obtained from Pantomics (OVC2281) and processed/stained by the Swanson Biotechnology Center (Cambridge, MA). Phospho‐p38 antibodies were diluted as recommended by the manufacturer.

### 2.4 Retrovirus transduction

For VSVG‐pseudotyped virus production, 293T cells were transfected using the calcium phosphate method (Clontech) using pMLP along with packaging and structural vectors VSVG and GAG/POL. Supernatants containing virus were then used to transduce target cells in the presence of 8 μg/ml polybrene for three rounds of infection. Successfully transduced cells were selected either with puromycin (3 μg/ml) for pMLP infected cells, or were sorted for GFP expression by flow cytometry for GFP‐Luciferase used to infect OVCAR8 cells for bio‐luminescent imaging and was a gift from B. Huang (MIT).

### 2.5 qRT‐PCR

For qRT‐PCR analysis, RNA was extracted from mouse tumor or cells using TRIzol reagent (Ambion) according to the manufacturer's instructions and 1 μg of total RNA was used for reverse transcription using the Superscript III First‐Strand Synthesis kit (Invitrogen) as per the manufacturer's instructions. For qPCR, data cDNA was amplified using SYBR green PCR mastermix (Applied Biosystems) according to the manufacturer's cycling conditions for 40 cycles on a Bio‐Rad C1000 Thermal Cycler. Data were analyzed using the delta‐delta Ct method as described previously and plotted as fold change versus control.^34^, ^35^ Primers were ordered from Invitrogen and/or IDT: Actin hsa Fwd gaaaatctggcaccacacc, Actin hsa Rev catactcctgcttgctgatc, MK2 hsa Fwd gaagtgcctgctgattgtca, and MK2 hsa Rev tcaaagagttgtggctggtg, GAPDH Fwd GAAGGTGAAGGTCGGAGTCAAC, and GAPDH Rev CAGAGTTAAAAGCAGCCCTGGT.

### 2.6 Western blot

Cells were lysed in RIPA lysis buffer containing protease and phosphatase inhibitor (Roche). Protein concentration was measured using BCA (Pierce) and cell extracts containing the same amount of protein were mixed with 6× reducing sample buffer, boiled at 95°C for 5 min, and subject to electrophoresis using standard SDS‐PAGE methods. For LICOR‐based blotting, proteins were transferred to nitrocellulose membranes (Biorad) and blocked with Odyssey blocking buffer for 1 hr. Primary antibodies were incubated overnight at 4°C followed by secondary antibodies conjugated with LICOR fluorophores, then scanned with a LICOR/Odyssey infrared imaging system (LICOR Biosciences). Band densitometries were quantified using ImageStudio. For ECL‐based blotting, proteins were transferred to methanol‐activated PVDF membrane (Biorad) and blocked with 5% nonfat dried milk for 1h. Primary antibodies were incubated overnight at 4°C followed by secondary antibodies conjugated with HRP for developing with ECL (Perkin Elmer).

### 2.7 Electron microscopy

0.125 nmol streptavidin‐gold (1.4 nm; Nanoprobes) and 0.5 nmol biotin‐modified siRNA (Ambion Silencer Negative Control No. 1) were reconstituted with peptide amphiphiles as described above and purified by dialysis (50 kDa) against ultrapure water at 4°C. TEM was carried out using a JEOL 2100 FEG instrument at an accelerating voltage of 200 kV. Samples were prepared by dropcasting particle suspensions onto a TEM grid with a QUANTIFOIL Holey Carbon Film (Electron Microscopy Sciences). Cryogenic transmission electron microscopy (TEM) was performed using a JEOL 2100 FEG instrument equipped with a Gatan 626 Single Tilt Liquid Nitrogen Cryo Transfer Holder. Vitrified samples were prepared on QUANTIFOIL Holey Carbon Films using a Gatan Cryo‐Plunge3 system.

### 2.8 Live‐cell confocal fluorescence microscopy

Imaging was performed using a Nikon 1AR Ultra‐Fast Spectral Scanning Confocal Microscope equipped with an environmental chamber providing temperature control. Caov3 cells were passaged onto 35 mm glass‐bottom culture dishes (MatTek, no. 0). Adherent cells were washed with DPBS and concurrently incubated with nanoplexes (50 nM, AllStars Negative Control siRNA, Alexa Fluor 488; Qiagen), LysoTracker Deep Red (50 nM, Thermo Fischer), and Hoechst 34580 (10 μg/ml, Thermo Fischer) in Opti‐MEM media at 37°C for 1 hr. Cell monolayers were then washed in DPBS and imaged in HEPES buffer (10 mM, pH 7.4).

### 2.9 In vivo studies

Female NCr nu/nu and BALB/c mice (6 wks) were obtained from Taconic. Orthotopic OVCAR8 tumors were induced in NCr nu/nu mice following i.p. injection of 300 μL of DPBS cell suspensions containing 5 × 10^5^ viable cells/mouse as measured by trypan blue exclusion. Subcutaneous OVCAR3 tumors were induced in NCr nu/nu mice following s.c. injection of 200 μL of DPBS:Matrigel (50% v/v) cell suspensions containing 5 × 10^6^ viable cells/flank as measured by trypan blue exclusion. Tumors were treated after 4 weeks. siRNA accumulation was approximated using a Xenogen IVIS Imaging System (Caliper). Subcutaneous tumor‐bearing mice (n = 3) were intraperitoneally injected with nanoparticles (1 mg/kg, AllStars Negative Control siRNA, Alexa Fluor 647; Qiagen) dispersed in PBS. After 24 hr, mice were anesthetized with isoflurane and imaged (640/700 nm ex/em). Recovered fluorescence from the rear flank and whole animal was quantified using the region‐of‐interest analysis package in Living Image (Perkin Elmer). siRNA accumulation in orthotopic tumors was qualitatively imaged using a Typhoon Variable Mode Imager (ex 488, em 555/20 nm; ex 633, em 670/30 nm). Bioluminescence imaging was performed on a Xenogen IVIS Imaging System at 10 min following i.p. injection of 300 μL of 15 mg/mL D‐luciferin potassium salt (Perkin Elmer) diluted in DPBS.

Tumor samples for flow cytometry were excised, minced using a scalpel, and dissociated in 2 mL of collagenase I (Gibco, 600 μg/mL) in HBSS containing 3 mM CaCl_2_ for 18 hr at 37°C with shaking. Suspensions were filtered (40 μm), centrifuged, and washed twice in HBSS, then fixed in 4% formaldehyde/PBS for 30 min at 37°C, prior to centrifugation and dilution in 1% BSA/PBS. Samples were analyzed on a BD LSR II flow cytometer.

Histological tissue samples were excised, immediately fixed in formalin for 72 hr, and stored in 70% v/v ethanol prior to paraffin‐embedding, processing, and immunostaining by the Swanson Biotechnology Center (MIT). Primary antibodies were diluted per the manufacturer's recommended conditions. Slides were scanned using a Leica Biosystems Aperio Digital Pathology Slide Scanner.

Serum samples were obtained at the study endpoints using BD microtainer SST tubes and frozen at −80°C prior to analysis by MIT's Comparative Pathology Laboratory. Ascites samples were obtained at the study endpoints and cells/debris were removed by centrifugation (800 g, 10 min). Samples were frozen at −80°C prior to MCP‐1 analysis by Eve Technologies (Calgary, AB, Canada).

Mice received cisplatin (Sigma‐Aldrich) and paclitaxel (LC Labs) as indicated (i.p., 300 μL/mouse). Paclitaxel was solubilized in Cremaphor EL:ethanol (50% v/v). Cisplatin was solubilized in DPBS. Total Cremaphor EL : ethanol content was  < 1% v/v of the injection volume.

Study endpoints included poor body condition (BC < 2), abdominal distension that impeded mobility (as determined by DCM veterinary staff), as well as bioluminescence flux that exceeded burden corresponding to 1,000 mm^3^ of excisable tumor volume. These experiments were approved by the Massachusetts Institute of Technology Committee on Animal Care.

## 3 RESULTS AND DISCUSSION

### 3.1 Rational design of an RNA‐peptide nanoplex

To achieve in vivo RNAi, we engineered a peptide‐based nanoplex composed of end‐functional NCA polymers. Ring‐opening polymerization of benzyl l‐aspartate NCA and subsequent deprotection or methylation, yielded three distinct amphiphilic peptides (Figure 1a, Supporting Information Figure S1a). Here, hydrophobic tails of all three amphiphiles were composed of poly(benzyl l‐aspartate), while hydrophilic head groups comprised either a monovalent quaternary ammonium salt, a short linear poly(ethylene oxide) chain, or a zwitterionic alanine moiety. The cationic ammonium group was included to facilitate electrostatic complexation with the phosphate backbone of siRNA and a 5 kDa poly(ethylene oxide) block was designed to facilitate steric shielding from endo‐ and exonucleases in the blood. A zwitterionic head group, a moiety commonly employed in so‐called “helper” or “fusogenic” lipids,^36^ was included here to facilitate entry of siRNA into the cytosol following cellular uptake, where bound nucleic acids can interact with proteins‐associated with the RNA‐induced silencing complex (RISC).

Peptide nanoplexes were formed by a modified ethanol dilution^37^ method (see Methods for details). Peptide‐siRNA nanoplexes obtained at an N‐to‐P ratio of 1 were 90 ± 11 nm in hydrodynamic diameter and nominally neutral in surface charge (−4 ± 4 mV) with relatively narrow polydispersity, as measured by dynamic light scattering (Figure 1b,c). Oligonucleotide loading and encapsulation efficiency were 0.48 wt% and 75 ± 10%, respectively, as measured by RiboGreen assay. Cryogenic transmission electron microscopy (cryo‐TEM) of hydrated nanoplexes supported these size measurements and further indicated a spherical morphology (Figure 1d, Supporting Information Figure S1b). Electron microscopy of dehydrated nanoparticles formed using siRNA labeled with 1.4 nm gold nanoparticles further suggested that oligonucleotides were spatially distributed evenly throughout a complex core (Figure 1e, Supporting Information Figure S1c). This morphology is presented in contrast to a more traditional micellar or lamellar core‐shell structure in which siRNA complexation is expected to be limited to the outer surface or leaflet bilayer. This less organized but uniform morphology suggests strong interactions between siRNA and the charged and hydrophobic blocks to yield a blended core nanoparticle which we hypothesize enables more facile intracellular release of siRNA. The critical aggregation concentration of these peptide‐siRNA suprastructures was 18 ± 7 µM as measured by pyrene fluorescence assay, approximately 2.3‐fold greater than the estimated minimum initial blood concentration following systemic (e.g., intravenous) administration at 1 mg/kg siRNA in mice (Figure 1f). The resulting nanoparticle system was designed for intraperitoneal administration in which nanoparticles would be more directly exposed to tumor sites; therefore, in these initial studies, a specific tumor cell targeting ligand was not incorporated.

In vitro, nanoplex‐transfected OC cells exhibited diffuse, cytoplasmic fluorescence, as well as puncta colocalized with acidic organelles (Lysotracker), when fluorescently labeled siRNA was complexed and delivered to cells in culture (Figure 1g, Supporting Information Figure S1d). In vivo, peptide nanoplexes targeted subcutaneous ovarian tumor xenografts with high efficiency following systemic (intraperitoneal) administration of control siRNA labeled with a near‐infrared fluorophore (1 mg/kg siRNA). Fluorescence tomographic imaging indicated spatial penetration of siRNA throughout ovarian tumor xenografts (Figure 1h, Supporting Information Figure S1e) and corresponding epifluorescence imaging analysis found that 4.8 ± 0.8% of the injected dose (i.e., recovered fluorescence) co‐localized with the tumors (*t* = 24 hr, n = 4).

### 3.2 Loss of MK2 chemosensitizes ovarian tumor cells

**Figure 2:**
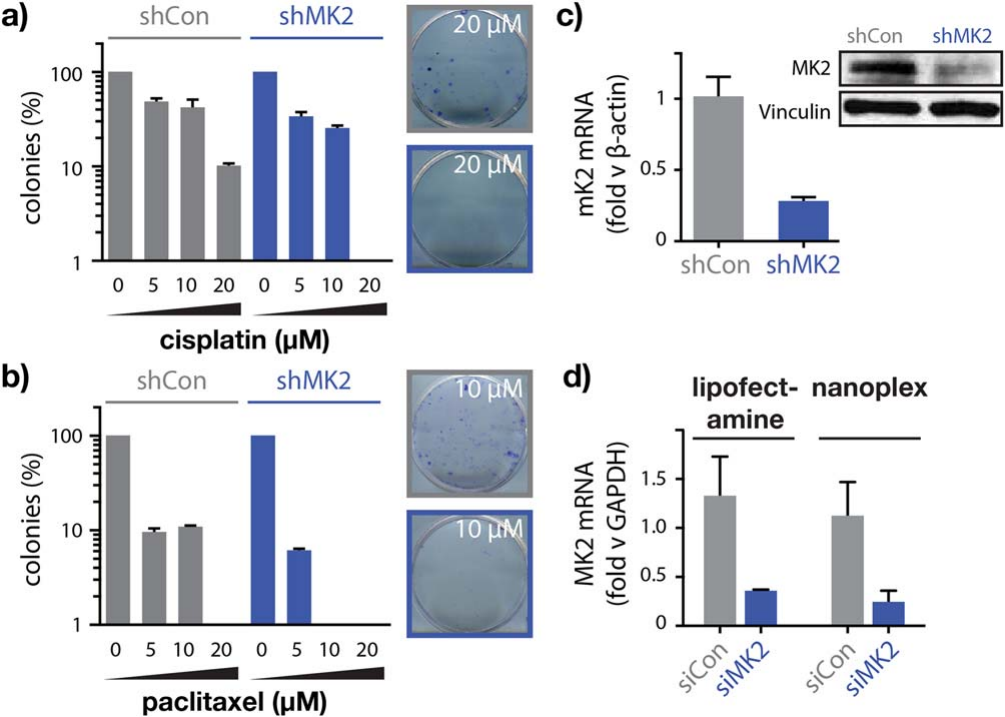
Loss of MK2 chemosensitizes ovarian tumor cells to platinum/taxane therapy in vitro. MK2‐dependent colony formation following pulsed exposure to (a) cisplatin or (b) paclitaxel relative to TOV‐112D cells transduced with control shRNA. (c) Real‐Time PCR (qPCR) and Western blot of MK2‐depleted cells from (a,b) demonstrating mRNA and protein downregulation. (d) RNA‐peptide nanoplex depletion *MK2* mRNA with efficiency equal to or better than commercial transfection reagents (i.e., Lipofectamine RNAiMAX). (d) COV362, 10 nM, 72 hr. Error represents *SEM* of three replicates

Having established a suitable platform for in vivo RNAi, we next sought a therapeutic target that was not currently druggable using small molecules, and whose functional silencing could improve treatment outcomes in patients currently receiving frontline chemotherapy for OC. We previously showed that pairwise loss of p53 and MK2 was synthetically lethal in chemotherapy‐treated, non‐small cell lung tumors;^28^ however, at present it was unclear to what extent these findings extend to other tumor types. We hypothesized that an MK2‐dependent DNA damage response would likewise be required for survival in chemotherapy treated ovarian tumors that are genotypically characterized by loss‐of‐function p53 mutations. Indeed, we found that activation of MK2's upstream signal effector, p38, was significantly enriched in serous ovarian tumors relative to matched normal tissues (Supporting Information Figure S2) and that clonogenicity of p53‐mutant ovarian tumor cells (TOV112D) was dramatically diminished following shRNA‐mediated MK2 depletion and platinum or taxane treatment in vitro (Figure 2a–c, Supporting Information Figure S3). Together, these findings provide strong support that loss of MK2 can chemosensitize p53‐mutant ovarian tumors to frontline therapeutic interventions for the disease (i.e., platinum/taxane doublet therapy). In vitro, we also observed nominally improved MK2 silencing efficiency from RNA‐peptide nanoplexes compared with commercial cationic lipid‐based transfection reagents (i.e., Lipofectamine RNAiMAX) at equimolar siRNA concentrations (COV362, 10 nM, 72 hr; Figure 2d), demonstrating efficient cytosolic delivery relative to known, non‐viral delivery vectors.

### 3.3 Peptide nanoplexes deliver siRNA to mouse models of OC

**Figure 3:**
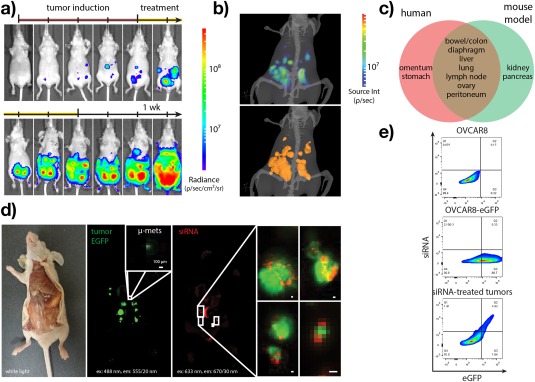
Nanoplex‐mediated siRNA delivery in an orthotopic mouse model of high‐grade serous ovarian cancer. Representative growth of orthotopically implanted OVCAR8 tumors as monitored by (a) bioluminescence imaging and (b) diffuse light imaging tomography (DLIT). (c) Anatomic sites of OVCAR8 tumor dissemination observed relative to the human disease. (d) Spatial co‐registry between siRNA (red) and tumor (green) fluorescence at 24 hr following nanoplex‐mediated siRNA delivery as measured by fluorescence imaging. (e) In vivo siRNA delivery efficiency as measured by flow cytometry of parental cells (top), GFP‐transduced cells (middle), and excised tumor tissues (bottom). Double positive cells in (e) represent 77 ± 5% of the GFP^+^ single‐cell population following nanoplex‐mediated siRNA delivery (1 mg/kg, i.p, 24 hr). OVCAR8 cells were retrovirally transduced to express enhanced green fluorescent protein (eGFP) and firefly luciferase (fLuc). Note that kinetics in panel (a) reflect average growth from independent biological replicates and that tumor localization over time may not necessarily coincide; 5 × 10^5^ cells/mouse. Time scale in (a) denotes growth kinetics over tumor induction and treatment (sham) phases. Image in (b) obtained at Day 42 post‐implantation. (d) AllStars negative control siRNA, 3′‐Alexa Fluor 647; 488/555 nm ex/em (eGFP), and 633/670 nm ex/em (siRNA). (e) AllStars negative control siRNA, 3′‐Alexa Fluor 488

In order to assess the therapeutic benefits of nanoplex‐mediated RNAi of MK2, we next established a mouse model of OC that faithfully recapitulates the genetic and histopathologic features of high‐grade serous disease. Shultz and coworkers^38^ recently identified a subset of OC cells that — in contrast to lines often employed — more closely resembles the copy‐number changes, mutations, and mRNA expression profiles presented by high‐grade serous ovarian tumors. We^39^ and others^40^, ^41^ later identified cell lines among these whose phenotypic and histopathological features, in vivo, further aligned with that of the human disease. In these studies, we selected the OVCAR8 cell line due to its relatively aggressive growth rate, strongly mesenchymal phenotype, ability to form intraperitoneal tumors with high‐grade histopathological features, and murine disease accompaniment by ascitic fluid accumulation. To facilitate longitudinal monitoring of tumor‐bearing mice, parental OVCAR8 cells were transduced with a retroviral vector expressing GFP and firefly luciferase. OVCAR8‐GFP‐fLuc cells formed excisable tumors approximately 28 days following intraperitoneal implantation into athymic NCr nude mice (5 × 10^5^ cells, Figure 3a) with ascites accumulation at 28–91 days post implantation. In all cases, tumors were spread throughout the abdominal cavity and seeded sites largely coincident with the human disease including the bowel/colon, peritoneum, liver, diaphragm, and mesentery (Figure 3b,c). OVCAR8 tumors displayed immunohistochemical markers of rapid proliferation (Ki67) and lineage‐specific markers of the ovarian surface epithelium (WT1, Supporting Information Figure S4). Although tumor lesions in untreated mice were typically intra‐abdominal, those without significant ascites accumulation often exhibited extraperitoneal (i.e., stage IV) disease at the study endpoints (median survival 59 days; Supporting Information Figure S5).

Following development of this orthotopic mouse model of high‐grade serous OC, we next examined the efficiency of systemic siRNA delivery to locally disseminated ovarian tumors. Using near‐infrared fluorophore‐labeled siRNA, we observed spatial co‐registry between GFP tumor fluorescence and siRNA fluorescence following systemic intraperitoneal administration of peptide nanoplexes (1 mg/kg siRNA; Figure 3d). Tumor dissociation and flow cytometric analysis revealed that 77 ± 5% of the GFP^+^ tumor cell population also stained positive for siRNA (Figure 3e). This high fraction of tumor cells that receive siRNA is encouraging as heterogeneous delivery could diminish chemosensitization effects in vivo. We hypothesize that the high efficiency with which peptide nanoplexes deliver siRNA here is mediated, in part, by direct intra‐abdominal tumor exposure following intraperitoneal administration. Interestingly, and in contrast to other tumor types, OC chemotherapy is frequently administered via intraperitoneal infusion as this method affords higher selectivity and improved tumor access compared with intravenous chemotherapy.^42^, ^43^ Thus, delivery of ovarian tumor‐directed nanoplexes by this method may be incorporated more readily into existing treatment protocols than in other tumor types.

### 3.4 Neoadjuvant siMK2 therapy improves OC treatment outcomes

**Figure 4:**
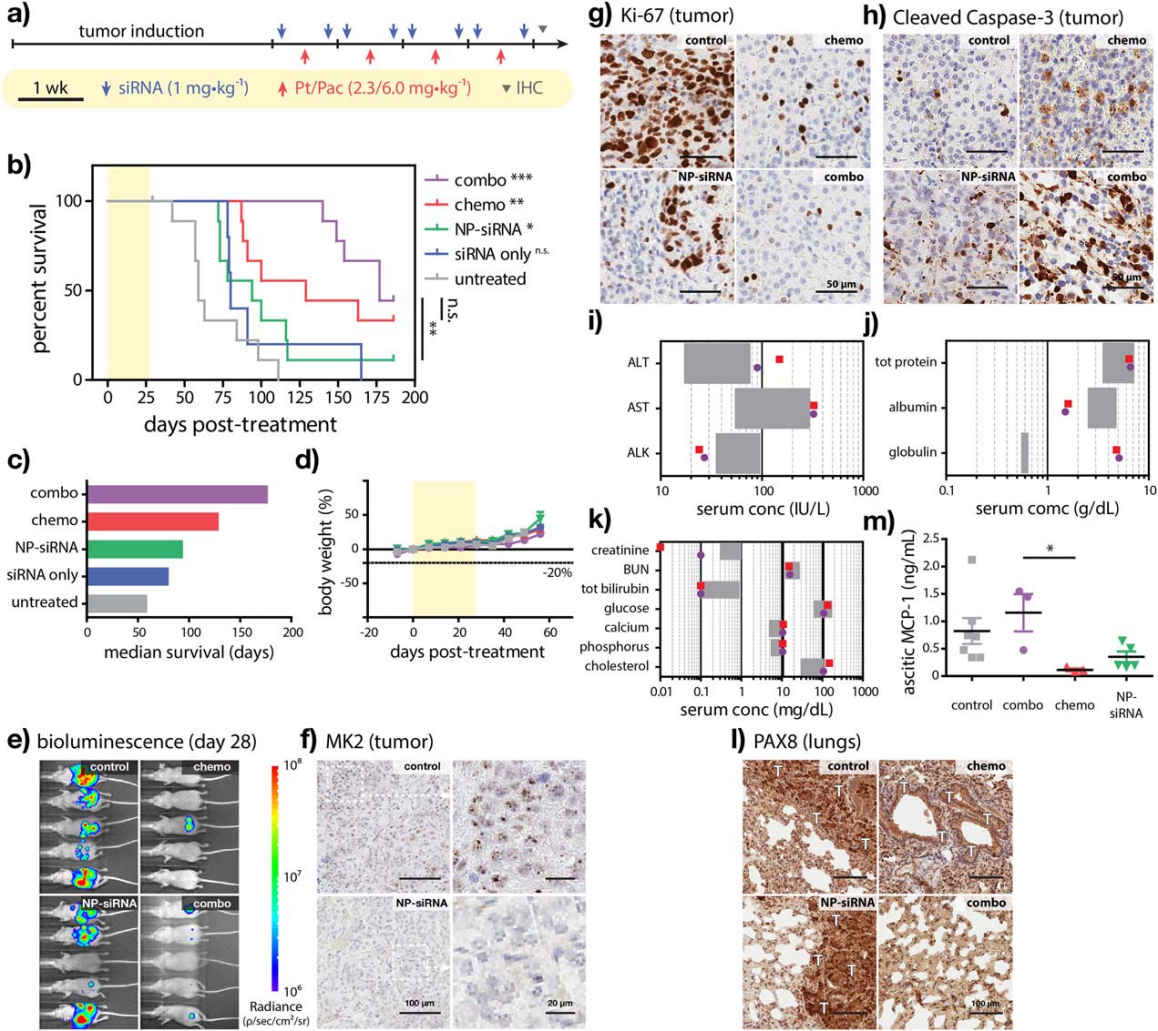
Nanoplex‐mediated RNA interference of MK2 sensitizes high‐grade serous ovarian tumors to platinum/taxane chemotherapy in vivo and improves overall survival. (a) Treatment schedule and (b) Kaplan–Meier survival curves for OVCAR8 tumor‐bearing mice receiving nanoplex therapy with or without concurrent cisplatin/paclitaxel therapy. Corresponding (c) median survival and (d) percent body weight change associated with varying treatment arms. (e) Tumor burden at treatment cessation (Day 28) as measured by bioluminescence imaging. Immunohistochemical staining of (f) parenchymal MK2, (g) Ki‐67, and (h) cleaved caspase‐3 at treatment cessation. (i–k) Serum biochemistry from mice receiving combined nanoplex/chemotherapy (purple) or chemotherapy alone (red) as measured at study endpoints. (l) Immunohistochemical staining of lung tissues obtained from mice at study endpoints displaying pulmonary metastases positive for a lineage‐specific marker of the ovarian surface epithelium (PAX8). (m) Ascitic MCP‐1 levels obtained from mice at study endpoints. (i–k) gray boxes denote normal reference ranges. (l) T denotes tumor regions. Error represents (d) mean ± *SEM* of 5–10 biological replicates and (m) mean ± *SEM* of 3–7 biological replicates. *p* < .05(*), *p* < .01(**), *p* < .001(***)

After demonstrating efficient in vivo delivery of siRNA to orthotopic, high‐grade serous ovarian tumor models, we next investigated treatment outcomes in OVCAR8 tumor‐bearing mice. In prior work,^44^ we observed robust silencing of MK2 over Days 2–5 following in vitro siRNA delivery. Thus, weekly treatment cycles consisted of siRNA (1 mg/kg, BIW) on Monday/Friday and cisplatin/paclitaxel (2.3/6.0 mg/kg, QW) on Wednesday (Figure 4a). Chemotherapeutic drugs, cumulative stoichiometric ratios, and excipients employed here were consistent with current National Comprehensive Cancer Network (NCCN) treatment guidelines^45^ for intraperitoneal OC chemotherapy (stages II–IV). A relatively modest dose of cisplatin was used in this case as OVCAR8 is a platinum‐sensitive^41^ cell line.

Following induction, OVCAR8 tumor‐bearing mice received platinum/taxane chemotherapy with or without concurrent nanoplex‐siRNA therapy for four weekly cycles, then were monitored for 180 days (Figure 4b). Although nanoplex‐siRNA monotherapy significantly improved median survival relative to untreated controls, outcomes were nominally less‐favorable than chemotherapy alone (94 vs. 129 days). In contrast, combined nanoplex‐siRNA and chemotherapy greatly improved treatment outcomes compared with chemotherapy alone, extending median survival by 37% (48 days, 177 days total) in the absence of acute toxicity as measured by fractional body weight loss (Figure 4c,d). Tumor burden as measured from bioluminescence images obtained at treatment cessation (Figure 4e) agreed well with observed trends in overall survival.

Immunohistochemical analysis of tumors excised at treatment cessation also agreed well with the corresponding treatments outcomes and 1.37‐fold increased survival times (Figure 4f–h). Mice receiving chemotherapy alone exhibited decreased tumor proliferation (Ki‐67) and increased apoptotic cell killing (cleaved caspase‐3). Parenchymal MK2 levels in tumors treated with nanoplex‐siRNA monotherapy were notably diminished, while tumor proliferation and apoptosis were generally comparable with chemotherapy‐treated tumors. Mice receiving concurrent siMK2 therapy and chemotherapy exhibited the most dramatic decreases in tumor proliferation and increases in cell killing.

Having shown that nanoplex‐mediated RNAi of MK2 chemosensitized ovarian tumors to frontline chemotherapy in vivo, we next examined differential pharmacodynamics that may have predicated improved treatment outcomes in mice receiving combined siRNA and chemotherapy. In prior work (unpublished), we found that repeated intraperitoneal dosing of nanoplex‐siMK2 (1 mg/kg siRNA, BIW) did not significantly affect serum biochemical markers of liver and kidney damage.^44^ At the study endpoints, we observed no significant differences in serum biochemistry from mice receiving chemotherapy alone or that with those receiving concurrent RNAi therapy (Figure 4i–k). Renal and hepatic function was impaired in both groups; however, we could not attribute differential survival to varying morbidity as measured from sera. We also examined lung tissues obtained from mice that reached study endpoints. Immunohistochemical staining of these tissues using an ovarian lineage‐specific marker (PAX8) found no observable pulmonary metastases in mice receiving combined siMK2 and chemotherapy, while multiple tumor lesions were observed in all other groups (Figure 4l). Moreover, we found that ascitic fluids from mice obtained at study endpoints were significantly enriched in monocyte chemoattractant protein 1 (MCP‐1, CCL2) following treatment with combined RNAi/chemotherapy versus chemotherapy alone (*p* = .0384; Figure 4m). Interestingly, ascitic MCP‐1 elevations were absent in mice treated with peptide nanoplexes, alone. Additional, independent studies are currently underway to better understand the molecular mechanisms by which loss of MK2 modulates tumor invasiveness and overall survival in OVCAR8 tumor‐bearing mice that receive platinum/taxane chemotherapy.

## 4 CONCLUSIONS

Peptide nanoplexes are a promising, modular drug delivery platform that can enable enhanced tumor accumulation of a wide range of therapeutics including small molecules, siRNA, plasmid DNA, and mRNA.^5^, ^15^, ^46^, ^47^ Here, we show that a novel polymer blend architecture consisting of three NCA peptide homologs can self‐assemble to form stable siRNA nanoplexes that silence a key DNA‐damage response pathway in p53‐deficient advanced ovarian tumors. Nanoplex‐mediated silencing of MK2 profoundly sensitized high‐grade serous ovarian tumors to concurrent platinum/taxane chemotherapy in vivo, improved overall survival by 37%, and decreased metastatic spread to the lungs without observable toxic side effects.

Given their peptide backbone and FDA GRAS (generally regarded as safe) designation of their amino acid metabolites, these structures could serve as uniquely biocompatible alternatives to conventional, non‐natural siRNA delivery vectors. Kataoka and coworkers, for example, have shown that chemotherapy‐appended NCA polypeptides are well‐tolerated in patients both in the United States^48^ and in Japan,^49^, ^50^, ^51^ and protein‐mimetic NCA polypeptides such as glatiramer acetate have been approved for clinical use in the United States since 1997. In contrast to peptides synthesized by solid‐phase techniques or those produced through recombinant expression, the RNAi delivery technologies described here could be manufactured both in large‐scale and with diverse (i.e., non‐natural) chemical functionality, potentially enabling more facile commercial development.

Therapeutic inhibition of MK2 also holds promise for a range of other disease indications beyond that demonstrated here in ovarian tumors. Duvall and coworkers, for example, have shown that polymer‐mediated delivery of peptide inhibitors of MK2 can block intimal hyperplasia and failure of autologous vein graft transplants in rabbits, a procedure commonly used for coronary and peripheral artery bypass in patients.^52^ There, inhibition of the p38/MK2 pathway‐mediated stress response induced following graft harvest/transplantation greatly decreased profibrotic response and corresponding graft failure. Inhibition of MK2 using small molecule tool compounds has also been shown to synergize with blockade of other cell cycle checkpoint kinases in Ras‐driven solid tumors, augmenting tumor cell killing in a p53‐dependent manner.^53^ The discovery of safe and specific small molecule inhibitors of MK2 for rheumatoid arthritis^54^ therapy is also an active area of industrial research. RNAi‐mediated silencing of MK2 may obviate the need for such development as small molecules targeting MK2 have historically exhibited off‐target toxic effects and limited selectivity. Given the promising results shown here, we anticipate that peptide nanoplex‐mediated RNAi may serve as a safe, specific, and effective neoadjuvant therapy that can sensitize advanced ovarian — and other solid — tumors to frontline cytotoxic chemotherapy.

## CONFLICT OF INTEREST

The authors are listed as inventors on intellectual property disclosures related to the content of this work.

## Supporting information

Additional Supporting Information may be found online in the supporting information tab for this article.

Graphical Table of Contents

Supplementary Material
