# Supplementary material for: RNA‐Peptide nanoplexes drug DNA damage pathways in high‐grade serous ovarian tumors
Source: Bioeng Transl Med. 2018 Jan 19;3(1):26–36. doi: 10.1002/btm2.10086 (PMC5773954; doi:10.1002/btm2.10086)
Supplement: Supplementary file 1 — Graphical Table of Contents [file BTM2-3-26-s001.docx]

Table of Contents (85 of 85 words)


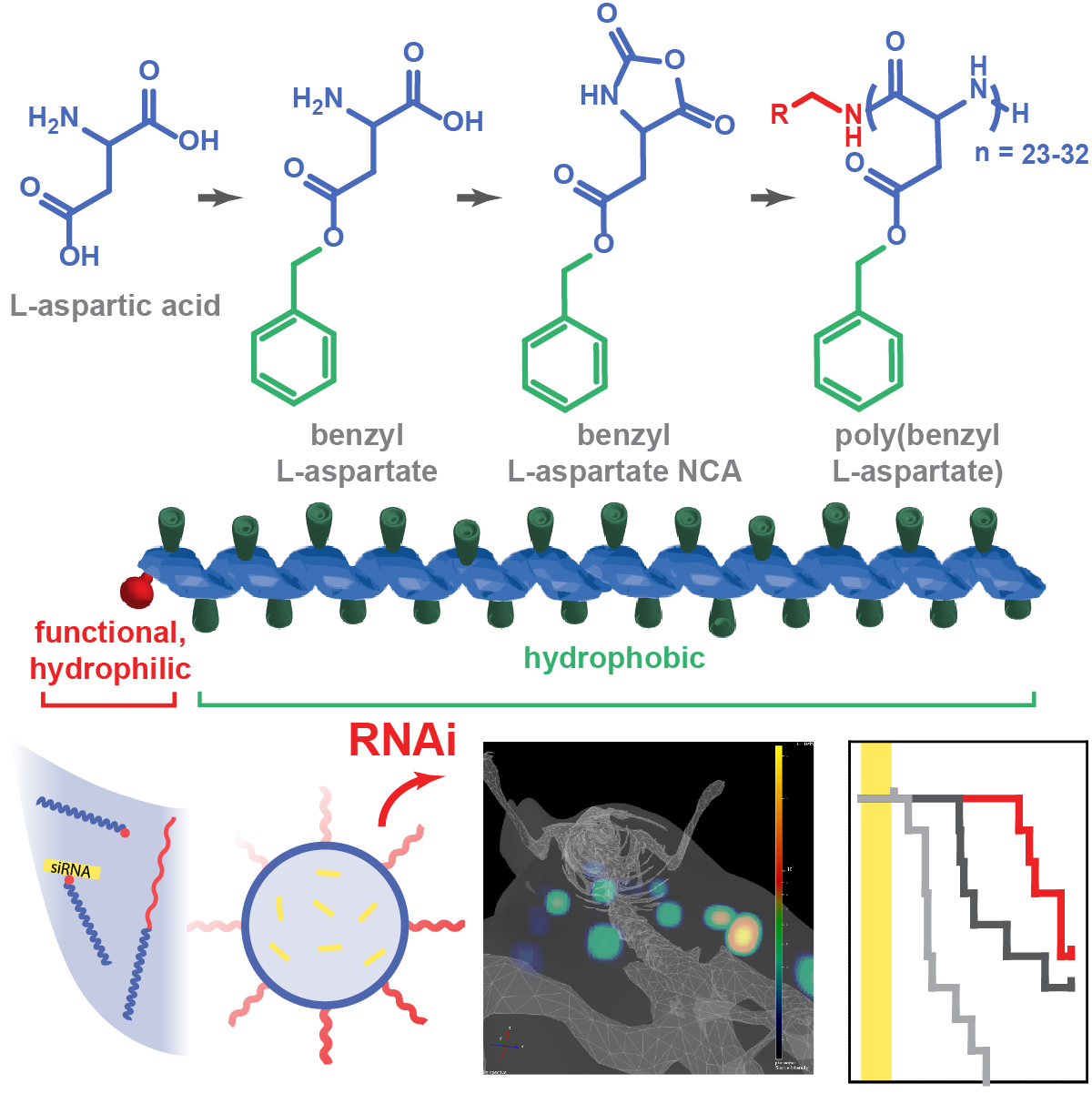


Most ovarian cancer patients either fail to respond or relapse following frontline chemotherapy. Treatments that block proteins involved in DNA damage responses induced by these drugs could thus sensitize tumors to initial treatment interventions. We recently identified one such protein, MK2 – considered undruggable by small molecules – and demonstrate here a polymer-based approach to silencing MK2 using RNA interference. By engineering a blend of synthetic peptides complexed with siRNA, we show efficient silencing of MK2 and profound sensitization to frontline chemotherapy in mouse models of ovarian cancer.
