# Supplementary material for: RNA‐Peptide nanoplexes drug DNA damage pathways in high‐grade serous ovarian tumors
Source: Bioeng Transl Med. 2018 Jan 19;3(1):26–36. doi: 10.1002/btm2.10086 (PMC5773954; doi:10.1002/btm2.10086)
Supplement: Supplementary file 2 — Supplementary Material [file BTM2-3-26-s002.docx]

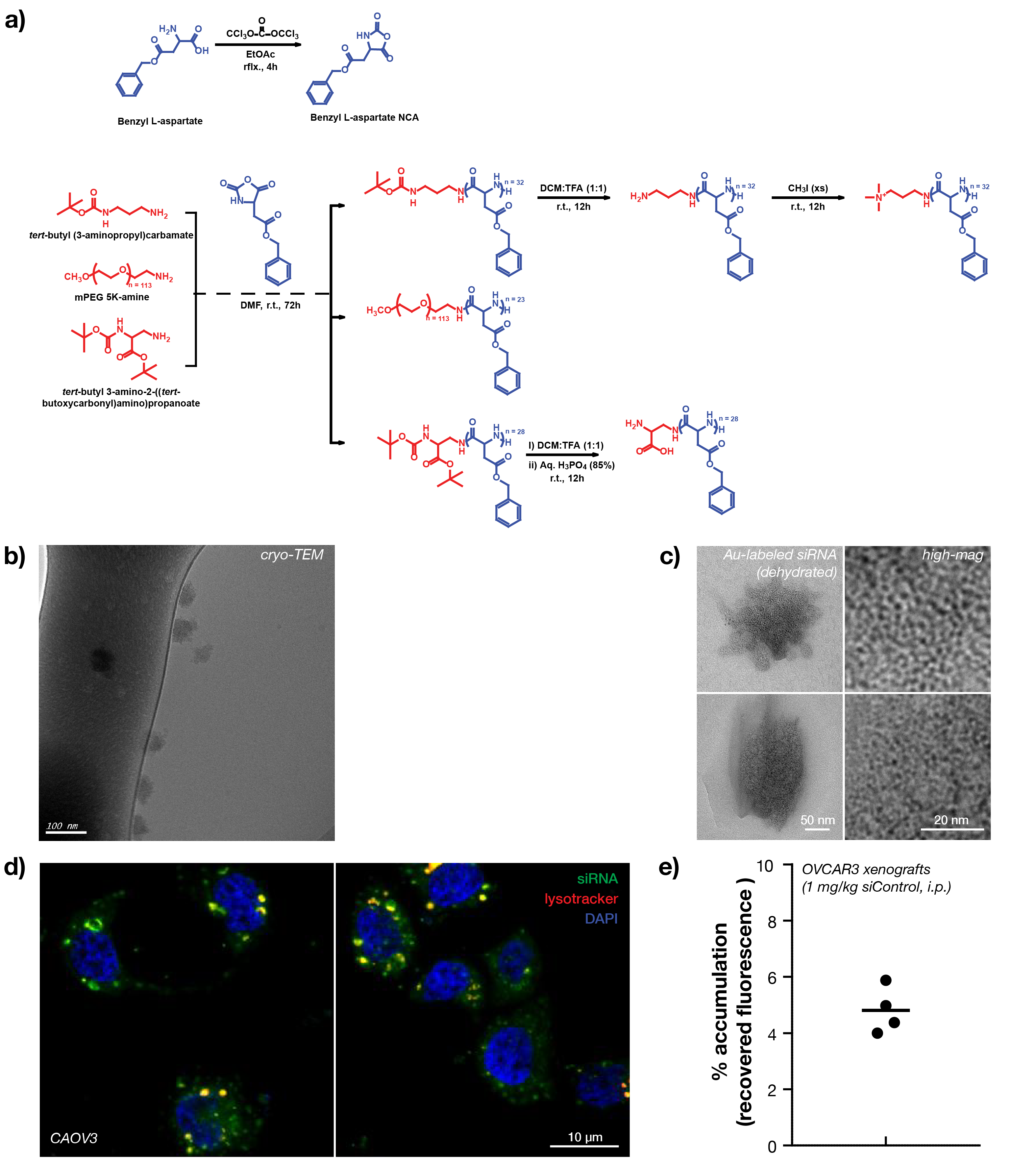


**Fig. S1. a)** Ring-opening polymerization of benzyl L-aspartate NCA and subsequent deprotection or methylation, yielded three distinct amphiphilic peptides. See Supporting Methods for details. **b)** Cryogenic transmission electron microscopy (cryo-TEM) of RNA-peptide nanoplexes in the hydrated state and **(c)** electron microscopy of dehydrated nanoparticles formed using gold nanoparticle-labeled siRNA showing homogeneous siRNA distribution throughout the ionic complex core. **d)** Live-cell confocal fluorescence microscopy of nanoplex-mediated cell transfection illustrating cytosolic delivery of siRNA (green) relative to acidic organelles (red) and nuclei (blue). **e)** siRNA accumulation within OVCAR3 hind-flank tumor xenografts following intraperitoneal administration. Data in (b-e) were obtained at an N-to-P ratio of 1. d) 20 nM siRNA (AllStars negative control, 3’-Alexa Fluor 488), vesicles (Lysotracker Red DND-99), and nuclei (Hoechst 34580) at 1 hr. e) 1 mg/kg siRNA (AllStars negative control, 3’-Alexa Fluor 647), 640/700 nm ex/em at 24 h. Error in (e) represents three biological replicates.

**
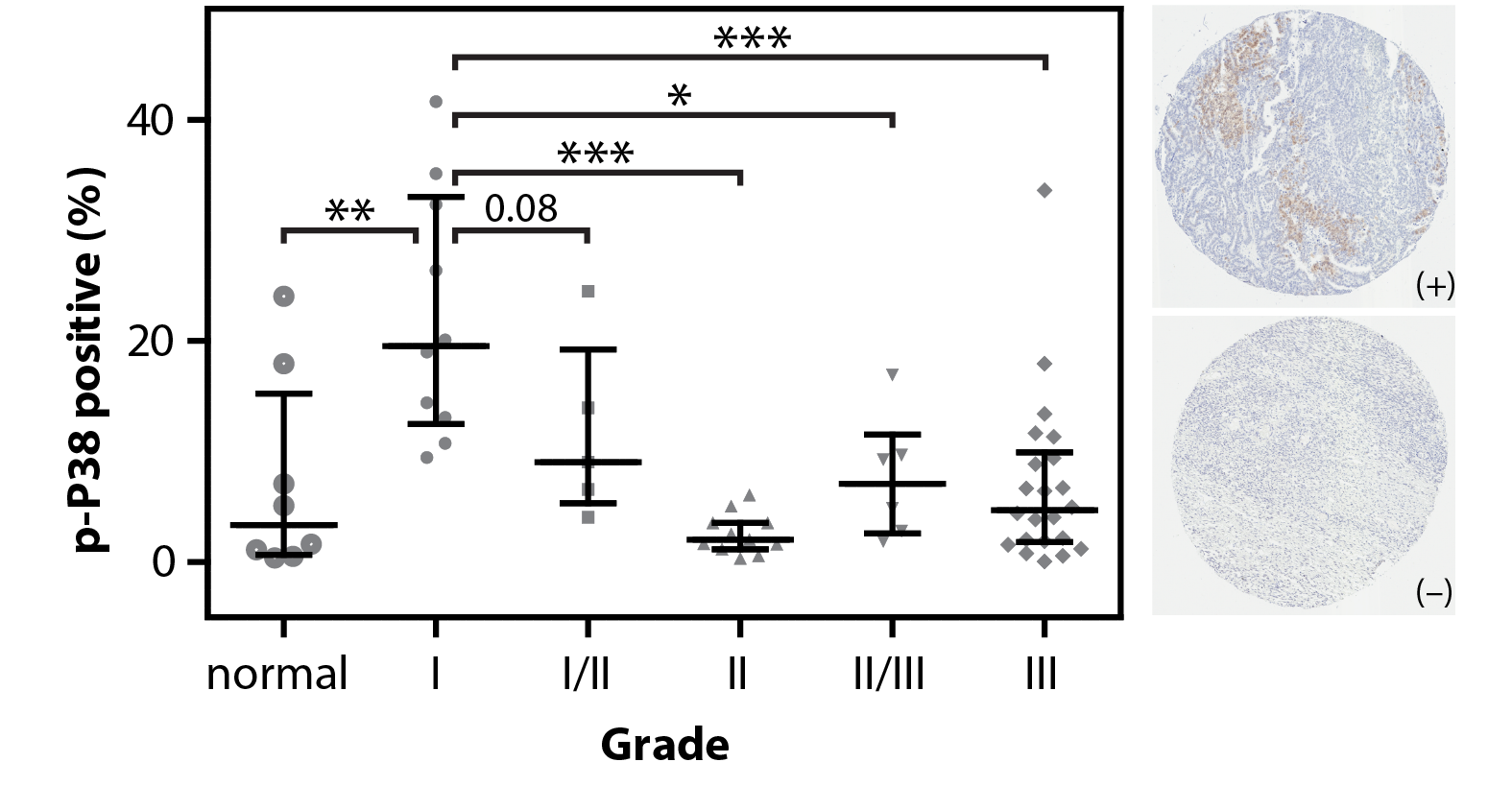
**

**Fig. S2. Upstream activation of P38/MK2 signaling is preferentially activated in ovarian tumors relative to matched normal tissues.** Immunohistochemical analysis of phospho-P38 staining in an ovarian tumor tissue microarray as scored using automated image analysis software. Bars represent median with interquartile ranges. P<0.05(*), P<0.01(**), P<0.001(***).

**
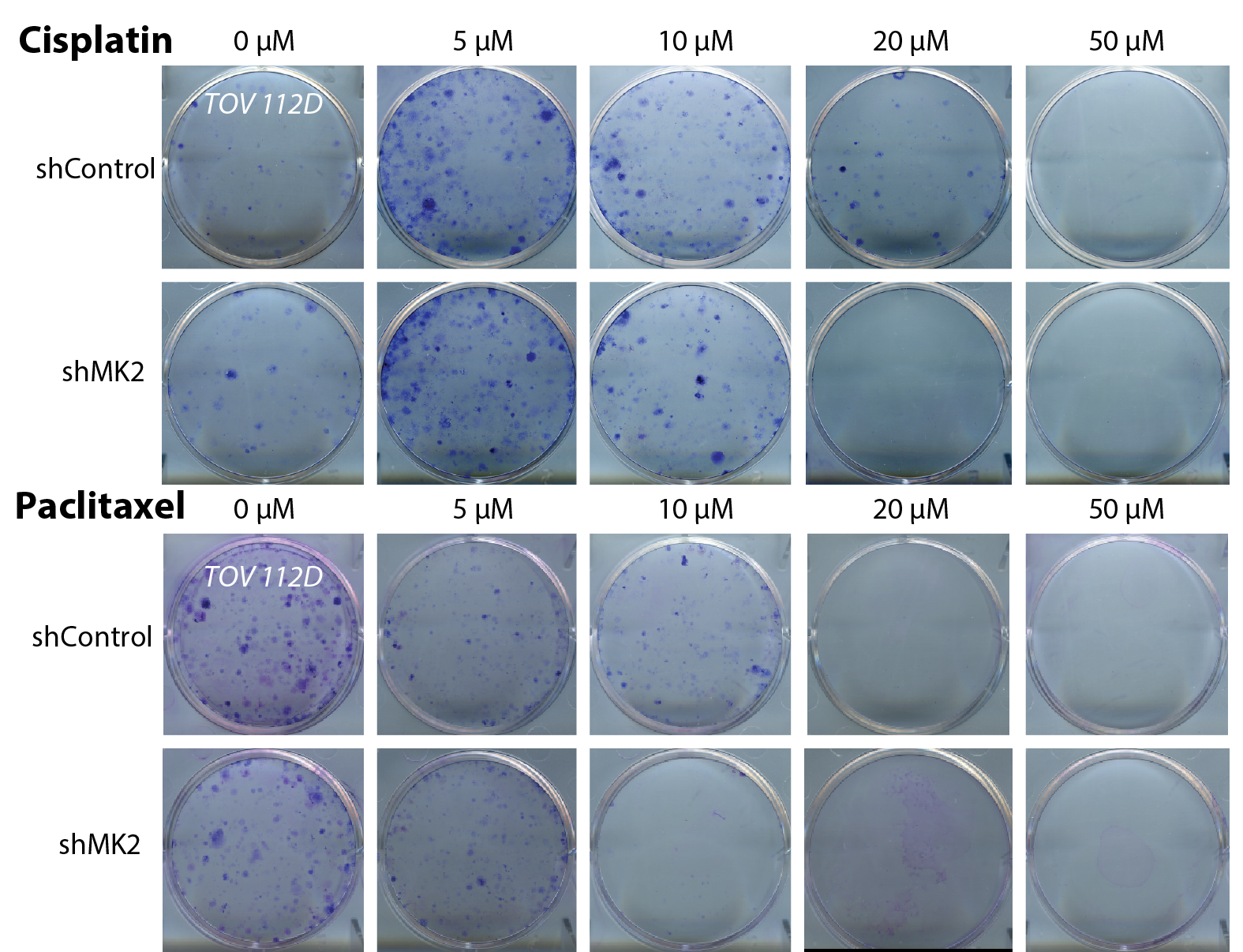
**

**Fig. S3.** MK2-dependent colony formation (as described in Figure 2) following pulsed exposure to cisplatin or paclitaxel relative to TOV-112D cells transduced with control shRNA.


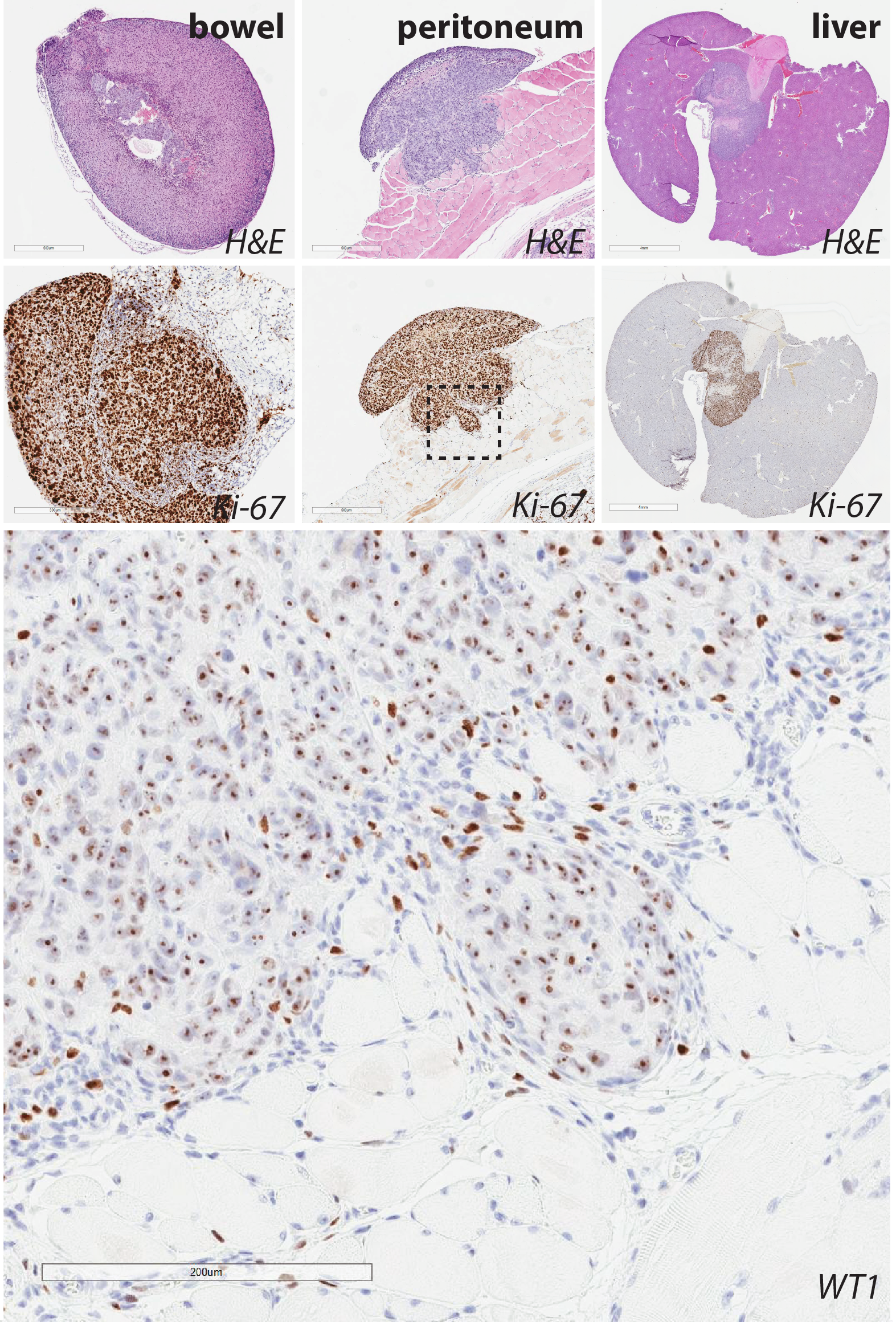


**Fig. S4.** Immunohistochemical staining of orthotopic OVCAR8 tumors explanted from the bowel, peritoneum, and liver of NCR nu/nu mice. Hematoxylin and eosin staining (top) and immunostaining of Ki-67 (middle). Wilms tumor protein immunostaining (bottom) from the indicated selected area.

**
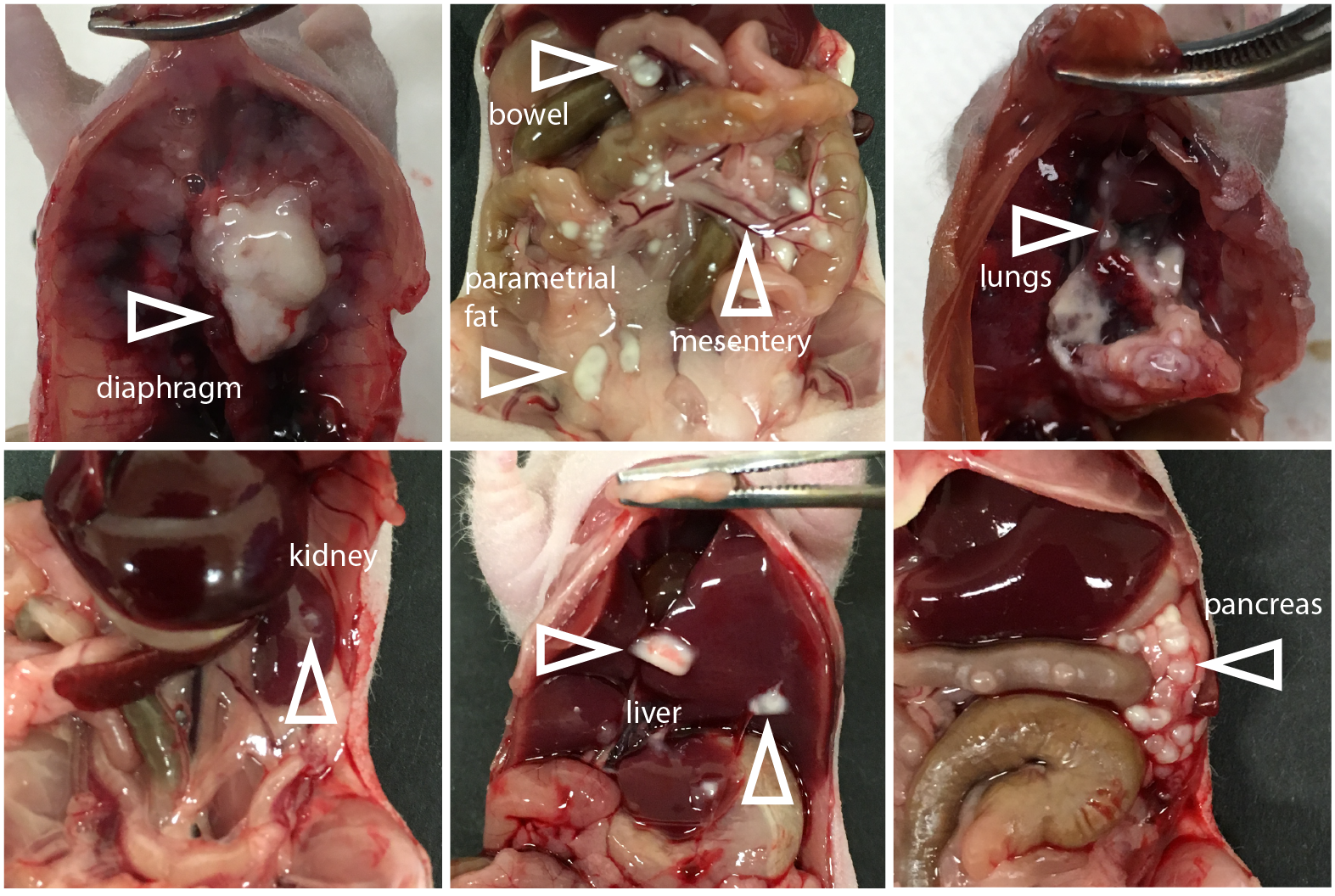
**

**Fig. S5.** Gross anatomy of lesions observed in OVCAR8 tumor-bearing NCr nu/nu mice.

**Supporting Methods.**

***Synthesis of Benzyl L-asparate N-carboxyanhydride (Benzyl L-aspartate NCA) (1):***

This synthesis was carried out according to well-established protocol described by Poche et al.^1^ Benzyl L-aspartic acid (4.5g, 2.09 equiv., 20.16 mmol) was dissolved in anhydrous ethyl acetate (90 mL) under inert atmosphere. The solution was bubbled with N_2_ gas, after which 2.85 g of triphosgene (9.6 mmol, 1.0 equiv.) was added to the solution. The suspension was heated to reflux (80 ºC) for 4h after which the solution became clear. The reaction solution was extracted with water, 0.5% sodium bicarbonate and brine. The organic phase was collected, dried over MgSO_4_. Reduction of solvent volume followed by addition of hexane precipitates the target product (**1**), benzyl L-aspartate NCA, which after drying gave an isolated yield of 70%.

***Synthesis of tert-butyl (3-aminopropyl) carbamate initiated poly(benzyl L-aspartate) homopolymer (2):***

tert-butyl (3-aminopropyl)carbamate (1.0equiv.) was dissolved in anhydrous DMF under (20 mL) N_2_ atmosphere to prepare the initiator solution. Benzyl L-asparate NCA (**1**, 50 equiv.) was dissolved separately in (5 mL) anhydrous DMF, and added to the initiator solution, and the reaction was allowed to run for 72h at room temperature. After the reaction period, the reaction mixture was added to ether and product was collected from diethylether solution by filtration. The precipitate was washed with water and lyophilized to yield tert-butyl (3-aminopropyl)carbamate initiated poly(benzyl L-aspartate) polymer (**2**) at 65% yield. ^1^H NMR (400 MHz, DMSO-d_6_, δ ppm): 8.18-8.16 (amide), 7.36-7.25 (bs, benzyl ester), 5.01, 4.65-4.49 (bs), 3.35 (bs), 2.82-2.80, 2.63-2.59, 1.32 (s, t-Boc). *M*_n_ (THF as eluent) = 7023.21 g/mol, PDI = 1.12, DP_n_ = 32, based on amino acid units.

***Deprotection of the BOC-group from tert-butyl (3-aminopropyl)carbamate initiated poly (benzyl L-aspartate) homopolymer:***

tert-butyl (3-aminopropyl) carbamate initiated poly(benzyl L-aspartate) polymer (**2**) was suspended in DCM:THF (1:1) mixture and stirred for 24h. ^1^H NMR was checked periodically to confirm complete removal of tBOC group from the homopolymer. The solvent mixture was evaporated and the product was washed with water and lyophilized to yield amine terminated poly(benzyl L-aspartate) homopolymer (**3**). ^1^H NMR (400 MHz, DMSO-d_6_, δ ppm): 8.18-8.17, 7.36-7.30 (bs, benzyl ester), 5.01, 4.63-4.25 (bs), 3.35 (bs), 2.87-2.79 (bs), 2.63-2.50 (bs).

***Quarternization of amine terminated poly(benzyl L-aspartate) homopolymer:***

Amine terminated poly(benzyl L-aspartate) (**3**, 1.0 equiv.) was dissolved in a mixture of acetonitrile and DMF (9:1, 10 mL). Ethyl bromide (1.1 equiv.) was added to the solution. The solution was refluxed for overnight, and the solvent volume was removed in a rotary evaporator. The condensate was precipitated in water and lyophilized to yield the quarternized homopolymer (**4**)

***Synthesis of α-methoxy-ω-amino-poly(ethylene glycol) (MeO-PEG-NH_2_, 5 kDa) initiated poly(benzyl L-aspartate) block copolymer (5):***

α-methoxy-ω-amino-poly(ethylene glycol) (1.0 equiv.) was dissolved in anhydrous DMF in a shlenk flask under N_2_ atmosphere. To this solution was added 50 equiv. of BLA-NCA, dissolved separately in anhydrous DMF. The reaction solution is allowed to run for 72h at room temperature, after which it was precipitated in diethylether. The precipitate was washed two times in water and lyophilized to yield poly(ethylene glycol)-b-poly(benzyl L-aspartate) copolymer in 73% yield. ^1^H NMR (400 MHz, DMSO-d_6_, δ ppm): 7.23 (bs, benzyl ester), 5.01 (bs), 4.63-4.61 (bs), 3.34 (-CH_2_-CH_2_-O-), 3.32 (-OCH_3_), 2.84-2.82, 2.60-2.58. *M*_n_ (THF as eluent) = 10,125 g/mol, PDI = 1.01. DP_n_ = 23.0 (based on amino acid units).

***Synthesis of tert-butyl 3-amino-2-((tert-butoxycarbonyl)amino) propanoate initiated poly(benzyl L-aspartate) homopolymer (7):***

In a dry shlenk flask, tert-butyl 3-amino-2-((tert-butoxycarbonyl)amino) propanoate (**6**, 1.0 equiv.) was dissolved in anhydrous DMF under N_2_ atmosphere. BLA-NCA (50 equiv.) was dissolved in the same solvent in a separate reaction vessel, and added to the shlenk flask containing the monomer **7.** The reaction was allowed to run for 72h at room temperature. After specified time period, the reaction solution was condensed, and precipitated in diethyl ether, washed with water (2X) and lyophilized to yield tert-butyl 3-amino-2-((tert-butoxycarbonyl)amino) propanoate initiated poly(benzyl L-aspartate) homopolymer (7). To synthesize the zwitterionic PBLA homopolymer **8**, deprotection of the BOC-carbazate of **7** was achieved by suspending the protected polymer in the mixture of DCM: TFA (1:1) for 12h at room temperature and the deprotection of BOC group was followed by ^1^H NMR. After achieving complete deprotection of the amine group, the BOC ester group was deprotected by treating the polymer with aq. Phosphoric acid (H_3_PO_4_, 85% w/v) in room temperature for 12h. ^1^H NMR (400 MHz, DMSO-d_6_, δ ppm): 8.17-8.13 (amide), 7.28-7.27 (bs, benzyl ester), 5.01, 4.63-4.61 (bs), 3.35 (bs), 2.85-2.82, 2.55-2.50. *M*_n_ (THF as eluent) = 6211.2 g/mol, PDI = 1.09, DP_n_ = 28 (based on amino acid units).

References

1. Poché, D. S.; Moore, M. J.; Bowles, J. L., An Unconventional Method for Purifying the N-carboxyanhydride Derivatives of γ-alkyl-L-glutamates. *Synth. Commun.* **1999,** 29, (5), 843-854.
